# New Perspectives on Risk Stratification and Treatment in Patients with Atrial Fibrillation: An Analysis of Recent Contributions on the *Journal of Cardiovascular Disease and Development*

**DOI:** 10.3390/jcdd10020061

**Published:** 2023-02-02

**Authors:** Giuseppe Boriani, Niccolò Bonini, Jacopo Francesco Imberti, Marco Vitolo

**Affiliations:** 1 Cardiology Division, Department of Biomedical, Metabolic and Neural Sciences, University of Modena and Reggio Emilia, Policlinico di Modena, 41124 Modena, Italy; 2 Clinical and Experimental Medicine PhD Program, University of Modena and Reggio Emilia, 41124 Modena, Italy

The medical approach to atrial fibrillation (AF) underwent a paradigm shift over time, evolving from considering AF as a simple arrhythmic phenomenon to a complex nosological entity [1,2].

Atrial fibrillation is the most common sustained arrhythmia in clinical practice, with a lifetime risk of one in three individuals aged ≥55 years old [3]. However, the epidemiological burden of AF may be underestimated since a significant number of AF cases may be asymptomatic and occasionally detected or require prolonged/continuous monitoring [4,5,6]. Asymptomatic AF, when clinically detected, even incidentally, is associated with the same risk of mortality and stroke/thromboembolic events as symptomatic AF [7], and this has promoted the screening of AF in order to detect asymptomatic AF and prescribe oral anticoagulants (OACs) in patients at risk [8].

The impressive evolution of technology in digital tools and wearables for checking cardiac rhythm has markedly widened the possibility of detecting AF [9,10], also as a consumer-initiated practice that should be addressed in medical consultations [11]. However, there are still grey zones for the widespread adoption of mobile-health devices, linked to limited digital literacy among elderly patients [12] and a lack of well-defined reimbursement policies [9,13].

Currently, the most recent European guidelines [14] advocate an integrated and holistic clinical management of AF patients through the Atrial fibrillation Better Care (ABC) pathway (i.e., “A” avoid stroke and starting anticoagulation when possible, “B”, better symptoms management and “C” cardiovascular and comorbidities optimization) [3,15,16,17]. Compared with usual care, adherence to the ABC pathway has been associated with a marked reduction in adverse outcomes in general AF patients and high-risk populations [17,18,19,20]. Additionally, given the complexity of AF, a structured characterization of the arrhythmia based on the 4S-AF scheme (Stroke Risk and Symptoms evaluation, Severity of Burden and Substrate of the arrhythmias) has been proposed [21,22].

Beyond the established benefit of OACs in patients at risk, in recent years, the clinical management of AF has also deeply focused on rhythm control interventions, mostly due to the evidence of randomised controlled trials (RCTs). Thus, the literature has concentrated on several risk factors and the interplay between comorbidities underlying AF development in the pre-clinical and clinical setting, especially in AF high-risk populations, aiming for better patients’ selection for rhythm control strategies and mitigating the risk of adverse outcomes [19,23].

Since 2020, the *Journal of Cardiovascular Development and Diseases* (JCDD) has significantly contributed to these central themes of AF. Among JCDD publications, 10% of the total articles were strictly related to AF, from basic science to RCTs. During these few years, there was a progressively increasing trend in AF publications, providing us with a rich insight into the topic.

As previously mentioned, much attention has been paid to the ultrastructural alteration of the left atrium (LA) in AF patients following the intriguing issue of atrial cardiomyopathy [24,25,26]. Several basic-science studies have investigated the micro-structural LA reproductions and the applicability of the new modality of catheter ablation in respect of such LA alterations [27,28,29,30]. The fetal AF theory was highlighted in some valuable papers enforcing the concept of the influence in AF predisposition both from the ambient, behaviour and lifestyle [31,32,33] and from the embryogenesis [34,35,36]/genetic pabulum [37].

In these years, many efforts have been made to consolidate the evidence regarding traditional pharmacological and non-pharmacological treatments in AF patients, including non-vitamin K antagonists (NOACs), LA appendage occlusion (LAAO), catheter ablation and promising treatments, such as sodium–glucose co-transporter (SGLT) inhibitors [38,39].

In the challenging group of patients with a high risk of bleeding, Chen et al. [40] elegantly showed no significant differences in thromboembolic and bleeding events between patients treated with NOACs and patients undergoing LAAO. In addition, Cepas-Guillen et al. [41] reported that low-dose apixaban after LAAO may be an excellent antithrombotic strategy to prevent the incidence of device thrombosis with a favourable safety profile compared with other antithrombotic therapies. 

Interesting evidence regarding pharmacological treatments in specific subgroups of patients with AF has been published [42]. Notably, recent data suggested that SGLT2 inhibitors may be associated with reducing the incidence of AF in HF patients [43].

Rhythm control strategies are becoming the first-line therapy for many AF patients [23,44]. Catheter ablation is one of the most improving techniques to treat non-permanent AF. An increasing amount of evidence on radiofrequency catheter ablation (RFCA), cryoballoon ablation (CB), and pulse-field (PF) ablation has been recently reported both in animal and human models [30,45,46].

Regarding RFCA optimization, Seidl et al. [47] found that a high-power short-duration ablation was comparable in terms of efficacy rates after one year to the conventional strategy approach. Another interesting analysis showed that unipolar electrogram-guided and lesion size index-guided RCFA were both effective and safe in patients with paroxysmal AF, but unipolar electrogram-guided might be more suitable for guiding RFCA [48].

Although RFCA has been the treatment of choice for many years, the use of CB has steadily increased, showing similar long-term outcomes [49,50]. Independently of the energy used, either radiofrequency or cryoenergy, several variables should be carefully evaluated, such as the duration of the application, position, stability and contact of the catheter, number of applications, etc., to produce the best transmural lesions [51]. In this perspective, a recent RCT reported no difference in AF recurrences between RF and CA [52]. Conversely, the NO-PERSAF trial highlighted how CB-CA vs. RF-CA led to shorter procedures and ablation duration, with less recurrence [53]. Gunkel et al. [54] compared the efficacy, safety, and characteristics of two cryoablation systems. The two systems (i.e., POLARx, Boston Scientific vs. Arctic Front Advance-Pro, AFA, Medtronic) were both effective and safe in AF patients, with some differences in procedure and fluoroscopy times as well as nadir temperatures. Comparing the mid-term efficacy and procedural outcomes of persistent AF patients with CB and robotic magnetic navigation (RMN), Li and colleagues [55] found that CB is generally equivalent to RMN-guided ablation. The PF-CA may be an additional resource in the future of AF ablation for its extreme specificity in inducing apoptosis in the myocardiocytes by electroporation. Although animal studies represented the most conspicuous part of the literature on this topic [30], some initial human experience found the procedure safe and relatively simple to learn [56].

Among the different types of CA presented above, the hybrid (surgical and endocardial) CA approach has been investigated, although less used and with a higher percentage of complications suggesting its use only in selected patients [57].

From a clinical standpoint, it is important to assess the efficacy of CA ablation, both in the short and long term, identifying possible predictors of recurrences. As a marker of procedure unsuccess, the presence of epicardial fat is associated with a higher recurrence risk after catheter ablation in AF patients [36,58]. Left atrial spontaneous echo contrast detected at the trans-esophagal echocardiogram (TEE) may also be a good predictor of the recurrence of AF after catheter ablation in patients with LA dilation [59]. Patient comorbidities are a crucial aspect of ablation success, also in terms of clinical outcomes. A multicenter analysis of AF patients treated with cryoablation found that patients with chronic kidney disease, even with mild to moderate reduction in renal function, were associated with a higher risk of AF recurrences [60]. Conversely, the procedural success and complication rates were similar in patients with normal, mildly reduced, or mild to moderate reduction in glomerular filtration rate [60]. A case-control study showed that obese patients did not have higher AF recurrence rates after CA compared to patients with normal body weight, suggesting that body mass index alone may not be a criterion for refusing catheter ablation [61]. Beyond catheter ablation’s efficacy in arrhythmia recurrences, some recent studies have explored the interesting association between AF and dementia and the possible role of rhythm control strategies in preventing cognitive decline [62,63]. For example, an interesting meta-analysis by Saglietto et al. [63] found that older patients with AF who underwent catheter ablation were associated with a nearly 50% reduction in dementia in a mid-term follow-up.

Lastly, a rising aspect is the risk of post-CA left atrial tachycardia (LAT), which may sometimes be more symptomatic than the initial AF [64,65]. Macro-reentrant tachycardia was the predominant electrophysiological mechanism of LAT. Interestingly, in most of the patients with macro-reentry LAT, there was a reconnection of at least one pulmonary vein [64].

The management of AF in particular subgroups of patients is undoubtfully a challenging clinical issue and deserves some consideration. Atrial fibrillation detected during infections or post-cardiac and non-cardiac surgery settings is one of the most controversial, with some uncertainty surrounding its best clinical practice [66,67]. On the one hand, Marazzato et al. [68], in an extended follow-up, found that postoperative AF (POAF) in cardiac surgery was not associated with an increased risk of mortality; on the other, a recent metanalysis found an increase of adverse outcomes in patients who underwent non-cardiac surgery [66] and the so-called “transient AF” had a two-fold higher risk of stroke and thromboembolism, suggesting that it should be not considered a benign entity without clinical implications at long-term. Regarding the prevention strategy of AF in the postoperative setting, Nomani and colleagues [69] summarized recent evidence of several RCTs and meta-analyses highlighting the possible efficacy of statins in POAF prevention, especially for atorvastatin. In addition, the Colchicine in Cardiac Surgery randomized clinical trial demonstrated the efficacy of short-term colchicine intake in preventing POAF after coronary artery bypass graft and/or aortic valvular replacement [70].

Interesting insights have also been recently reported in the setting of critically ill patients [71]. For example, in septic patients with AF, a low haemoglobin-to-red cell distribution width ratio was associated with an increased risk of all-cause death in-hospital, supporting the prognostic role of specific and routinely available biomarkers in the AF population [72,73,74].

Looking at the increased risk of AF incidence in particular populations, an observational study evaluated the possible early predictors of AF in post-stroke patients [75]. The study found that the supra-ventricular runs at the 7-day Holter-ECG were associated with a higher incidence of AF at three years of follow-up [75]. In addition, in a recent publication about heart failure with preserved ejection fraction (HFpEF) patients, women with AF vs. no AF had more abnormal structural and functional cardiac dysfunction with an increased risk of adverse cardiovascular outcomes independently of traditional risk factors and comorbidities [76]. In hypertensive patients, although several studies reported a clinical benefit of renin–angiotensin–aldosterone system inhibitors in reducing the incidence of AF, a comprehensive review highlighted that this benefit may be greatly reduced when AF diagnosis is already established, suggesting the need for the implementation of primary prevention strategies in patients at risk [77].

Overall, these studies claimed the necessity of structured screening programmes aimed at early AF detection and a rapid referral to a cardiologist for evaluation [10,14].

In conclusion, two leading concepts for the future perspective of AF research may be identified. First, AF-type substrate identification is becoming crucial. As mentioned above, there is an emerging focus on atrial cardiomyopathy, including the ultrastructural manifestation of the disease. In silico, computational and in vitro studies provided exciting insights for future clinical trials dedicated to AF ablation, intending to ensure more robust evidence. As per recent European Society of Cardiology guidelines [14] and the paradigm shift mentioned above, an in-depth substrate analysis of AF patients is necessary for the AF clinical evaluation within the 4S-AF approach [21]. The biological molecular data, histological and circulation biomarkers evaluation are becoming an essential part of the AF research panorama, striving for better comprehension of the previous and current mechanism of AF.

The second key message is that AF is not a lone disease. Clinical complexity is increasingly prevalent among patients with AF, thus requiring a holistic and structured approach to the patients [19]. Heart failure, hypertension and diabetes, chronic kidney disease, and cardiac structural and functional integrity should be carefully evaluated and managed with integrated care [78,79]. The clinical heterogeneity of AF patients often has a marked impact on outcomes and appears to complicate the planning of clinical trials. Moreover, the efficacy of some treatments in such a complex scenario may need to be revised in terms of evidence-based therapeutic proposals for AF patients. Considering the progressive ageing population and the burden of comorbidities, it appears desirable to maintain a virtuous circle from the experimental evidence, RCTs and “real-world” registries. The RCTs are the summit of the evidence pyramid providing the highest level of guideline recommendations, whose adherence could be verified in real-world disease-specific registries and large observational studies. This will result in a completer and more prosperous overview for future decisions on AF management, targeted to avoid the progression of AF and to improve patient outcomes [80].
